# Effect of a school-linked life skills intervention on adolescents' sexual and reproductive health skills in Guji zone, Ethiopia (CRT)—A generalized linear model

**DOI:** 10.3389/fpubh.2023.1203376

**Published:** 2023-10-23

**Authors:** Gobena Godana, Silesh Garoma, Nicola Ayers, Muluembet Abera

**Affiliations:** ^1^School of Public Health, Department of Reproductive Health, Jimma University, Jimma, Ethiopia; ^2^Federal Ministry of Health Ethiopia (Britain Origin), Addis Ababa, Ethiopia; ^3^Adama Hospital Medical College, Department of Public Health, Adama, Ethiopia; ^4^Dean School of Public Health, Jimma University, Jimma, Ethiopia

**Keywords:** adolescent, life skills, SRH, pastoral community, interventions

## Abstract

**Background:**

Although appropriate life skills are recognized as a fundamental right for adolescents and a critical component of health policy, they are often overlooked and rarely researched in pastoral communities. Life skills encompass the ability to adopt positive behaviors, which enable individuals to effectively deal with the demands and challenges of everyday life. This study aimed to evaluate the effectiveness of a school-linked life skills intervention program on adolescents' sexual and reproductive health skills in the pastoral community of Guji zone.

**Methods:**

A two-arm cluster randomized control trial with a pretest-posttest experimental design was conducted, involving the intervention group (*N* = 375) and the control group (*N* = 384). This study assessed the effect of a school-linked adolescent-friendly life skills intervention in comparison to the usual RH curriculum, used as a control arm. Pretest-posttest and posttest-posttest scores of the control group and trial groups were compared, and the data were collected using 27 self-administered questions. The collected data were analyzed using paired-sample independent *t*-tests and a generalized linear model to examine the relationship between the dependent and independent variables.

**Results:**

Data were collected from 759 adolescents in 15 intervention and 15 control clusters. The findings have shown that the proportion of mean life skills score was significantly higher in the intervention clusters than controls [(375) 70.49% vs. (384) 62.25%, *P* < 0.001 95% CI (0.06 and 0.1)]. Adolescents who were trained in school-linked life skills (β = 1.915, 95% CI: 1.411–2.418), were confident to make safe and informed decisions (β = 1.999, 95% CI: 1.562–2.436), and had life skills to deal with SRH issues (β = 1.66, 95% CI: 1.233–2.087) were significantly correlated with predicting the relevant life skills. The proportion of adolescents with SRH life skills increased from 384 (52%) at baseline to 375 (70%) at end line in the intervention group compared to 384 (60.31%) at baseline to 384 (62.31%) in control arms, respectively.

**Conclusions:**

The implementation of a school-linked life skills intervention program proved to have a significant effect on SRH life skills development. Furthermore, individual-level and behavioral-level variables were significant in explaining variability in life skills development within the pastoral community. Therefore, we recommend scaling up this intervention in all high schools.

**Trial registration:**

Trial registration PACTR202107905622610, registered on 16 July 2021.

## 1. Introduction

The latest scientific evidence strongly supports the idea that life skills intervention is essential for adolescents' life skills development, improved self-awareness, decision-making skills, and positive emotional growth through interactive learning and communicative skills (1–3). Thus, life skills are described as a multidimensional construct that includes the ability for adaptive and positive behaviors, enabling individuals to effectively cope with various demands and challenges. They provide individuals with strengths and capabilities that empower them to approach everyday life problems with a positive attitude and handle issues effectively with confidence (4, 5).

Various studies have proven the effectiveness of implementing life skills intervention in the general education context. For instance, a study conducted in the USA showed that ~66% of participants had lower odds of engaging in sexual activity compared to controls (6). A study in India showed that 91.5–95.1% of participants improved their awareness of various life skill domains (1, 7, 8). Magalhães et al. (9) and Gasol et al. (10) stated that 50.7% Portuguese and 48% Spanish adolescents who were exposed to life skills training had reported practicing the newly learned social skills. Similarly, Pradeep et al. (1) found that 91.6% of students in India reported improved awareness of life skills after participating in life skills training. Furthermore, studies conducted in Ethiopia by Malango and Hegena (11), Tesema et al. (12), and Baird et al. (13) showed that open parent discussions to develop SRH life skills had established positive longer-term impacts and increased student participation by 25.7, 24.5, and 8.2%, respectively (11–13). In North and East Ethiopia, a gender transformative life skills intervention program was conducted, demonstrating a significant improvement in knowledge of menstruation and its hygiene (14).

In sub-Saharan African countries, the lack of information about life skills contributed to the high risk of adverse SRH outcomes among adolescents. Hence, it is crucial to examine the gap in life skills training to address the practical needs of adolescents and understand the different co-existing factors in modern African societies (15). One possible reason for poor sexual and reproductive health outcomes among Ethiopian adolescents is the absence of life skills training (14). UN Care Ethiopia conducted life skills training for adolescents of UN staff in Ethiopia (16, 17); however, this training has yet to consider adolescents with visibly deficient gaps in life skills.

Since life skills training is essential for the development of adolescents, a school-linked friendly health intervention was designed and implemented within clustered high school sections. It helps adolescents to be more analytic and to make premeditated choices regarding their sexual health, resulting in fewer unintended pregnancies, reduced rates of sexually transmitted diseases, and improved interpersonal relationships (12). The school-linked life skills intervention program (LSIP) aims to develop appropriate SRH life skills in pastoral communities, helping adolescents acquire SRH awareness and essential life skills to address SRH issues and develop positive attitudes toward school-linked FHS among school adolescents in the pastoral community. Given that high schools are attended by nearly all adolescents, the school-linked friendly health intervention was implemented in 30 clusters of high school sections. This study aims to assess the effect of a school-linked friendly health intervention on adolescents' life skills development in the pastoral community of Guji zone, Ethiopia.

In addition, this study aims to compare the effectiveness of a school-linked friendly health intervention to regular reproductive health teaching programs on adolescents in pastoral communities in the East Guji zone, South Ethiopia.

## 2. Methods and materials

### 2.1. Study areas, participants, and period

**Figure 1:**
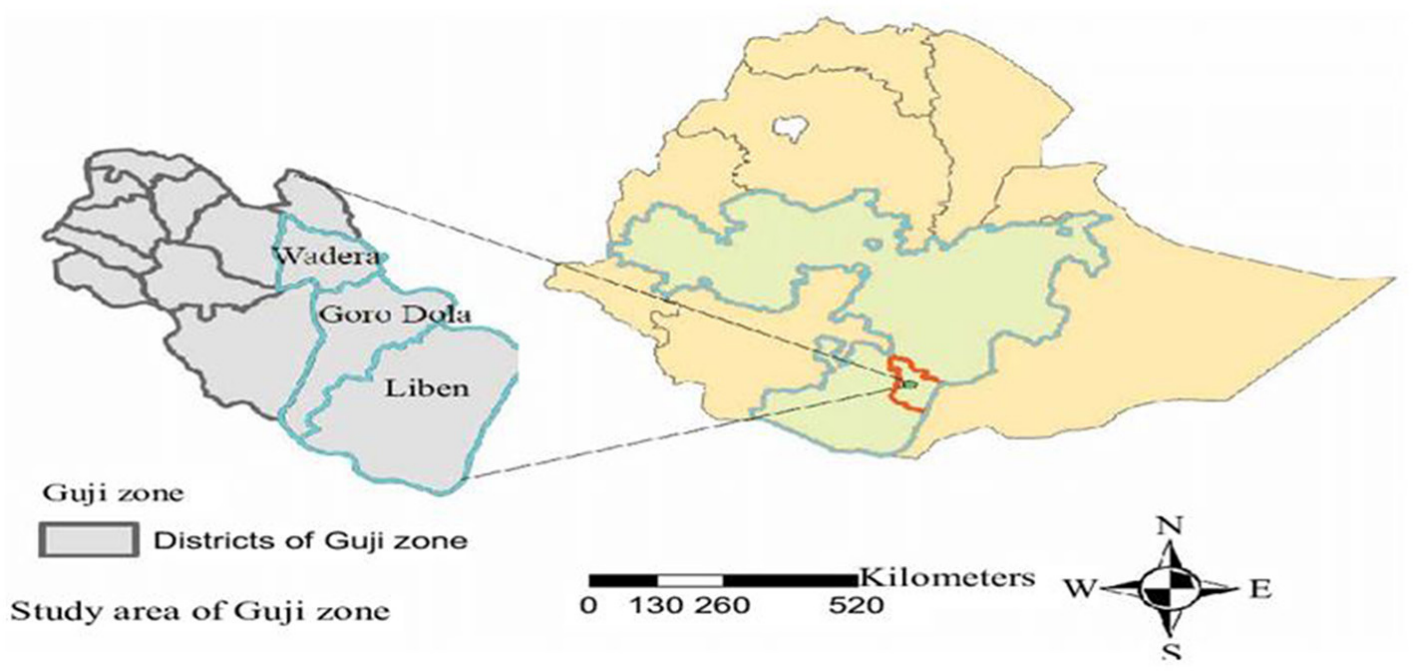
A map of a school-linked life skills intervention program in East Guji Zone.

This study was conducted in two pastoral districts, including Gorodola and Wadara, of the East Guji zone. East Guji zone is one of the administrative zones of the Oromia region in South Ethiopia. According to CSA 2007 Population Projection, the total target population for 2012 was 127,722 (18). The individuals who took part in the study were adolescents between the ages of 14 and 19 years, and they were attending Harekello and Wadara high schools. The study units were designated according to demographic characteristics and proximity. The designated adolescents and families were well versed in the research goal and provided informed consent beforehand. Lastly, two high schools and two preparatory schools, divided into 30 clusters, participated in the trial and control study. The source population were the middle- and late-aged adolescents (aged 14–16 years and 17–19 years, respectively) enrolled in selected schools. The study was conducted between November 2020 and May 2021 (Figure 1).

### 2.2. Study design

A school-linked cluster-randomized controlled trial study with a pretest-posttest design was used to evaluate the effect of school-linked LSIP on life skills development.

### 2.3. Sampling

**Figure 2:**
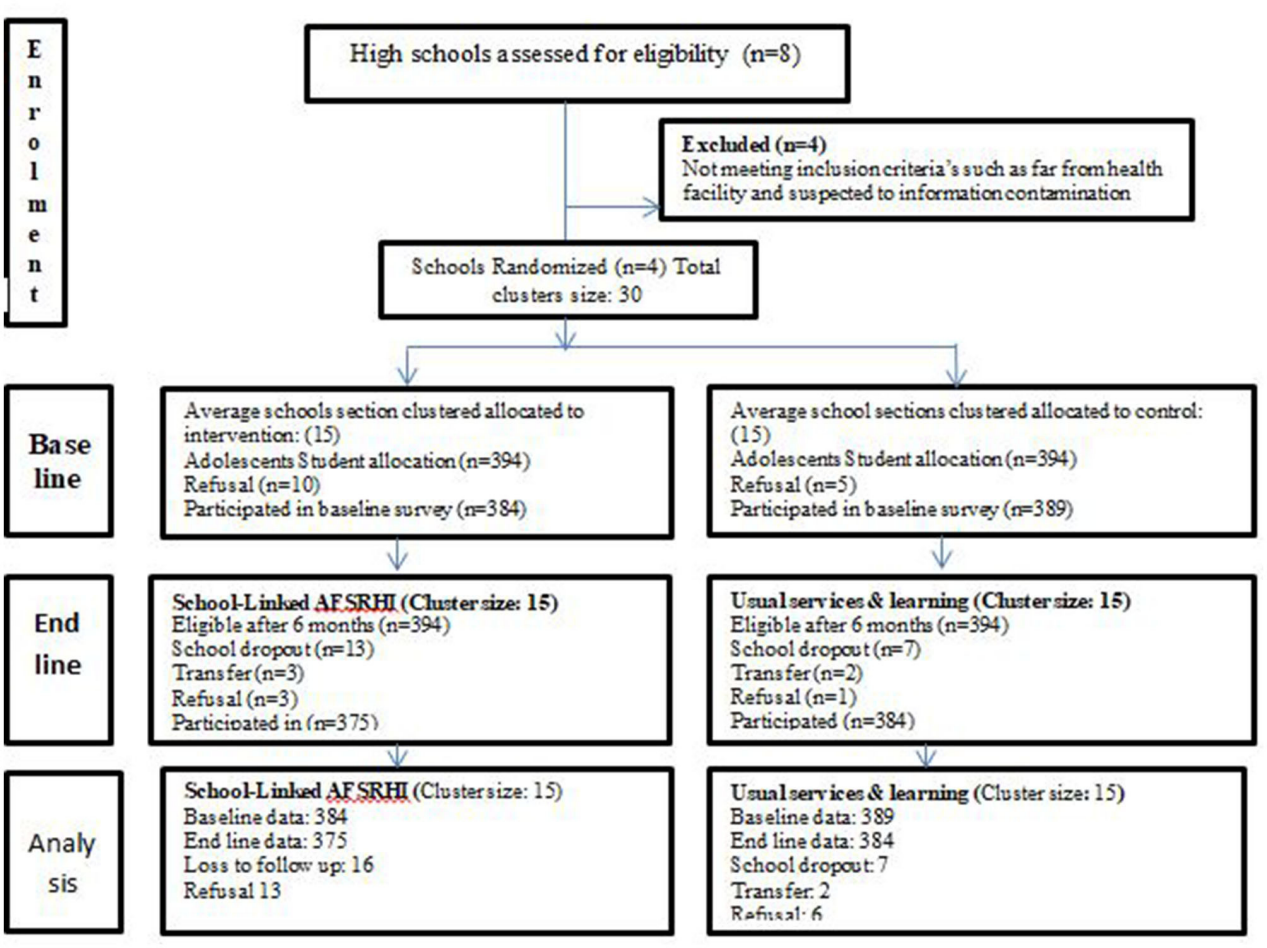
School-linked adolescents' friendly health intervention of life skill study clustering and sampling flow, East Guji zone, Ethiopia, from November 2020 to May 2021 (*N* = 1,532).

The sample size was determined according to the randomized controlled trials that recommended pairing equal-sized clusters based on sample size calculations for each cluster (19, 20). The study had 30 clusters available, with 15 clusters per arm, 95% CI, power 90%, and an intra-cluster correlation coefficient of 0.2 (21). The sample size was determined using a two-sample proportion comparison in Stata12 software, resulting in 179 observations for each treatment and control arm. We used assumption 2 as the design effect along with a 10% non-respondent rate (20, 22). This resulted in an average cluster size requirement of 30, leading to a total sample size of 788 adolescent students with 394 in the intervention group and 394 in the control group. Consequently, both baseline and end-line surveys included these 788 adolescents, who were in the grades 9–12 (see Figure 2).

#### 2.3.1. Randomization and sampling procedures

Gorodola and Wadara districts were selected from among the seven districts available in the East Guji zone. These districts were selected because both of them were part of a trial study aimed at enhancing adolescents' SRH life skills. The high schools within selected districts served as the cluster randomization units, and individual randomization was conducted among all high school sections. The sample size was randomly distributed between the intervention and control arm clusters in a 1:1 proportion (23).

#### 2.3.2. Eligibility criteria

The participants in the school-linked trial included adolescents, aged 14–19 years, who were enrolled in secondary schools within the designated districts. However, adolescents who were newly enrolled in designated schools and stayed in schools for a duration of <6 months were excluded from the study.

#### 2.3.3. Double blinding

The information about intervention was kept concealed from both trainees and data collectors. All adolescents were unaware that they were in the intervention or control arms. Although trainers were aware of the intervention packages, they were unaware of the specific schools receiving the intervention. Similarly, data collectors were unaware of the groups receiving the intervention, and the data were also collected by separate data collectors.

### 2.4. Descriptions of intervention

#### 2.4.1. Intervention arm I

To facilitate adolescents' life skills development, we conducted a facility-based baseline survey to identify adolescents' status of SRH life skills and categorize factors that predict appropriate SRH life skills development. The school-linked life skills intervention program addressed the identified gaps and factors that enhance SRH life skills development. Nearly all adolescents received school-linked life skills intervention program packages provided at three platforms such as age-relevant SRH education, life skill training, and reproductive health clubs. The experimental group participated in a school-linked life skills intervention program included 40 sessions, each lasting 20 min (totaling 26 h), from November 2020 until May 2021. The intervention targeted three categories of primary life skills outcomes: Life Skills for Self-Understanding and Self-Management, Life Skills for Knowing and Living with Others, and Life Skills for Dealing with Issues and Problems. Adolescents in the trial schools attended the training consisting of 20-min sessions, five times a month for 6 consecutive months.

#### 2.4.2. Control arm II

Adolescents in the control groups received the existing routine reproductive health unit in biology courses. However, they did not partake in any school-linked life skills intervention program packages. No detail about specific life skills categories were mentioned in regular sessions and there were no discussions on selected life skills topics in the reproductive health club.

#### 2.4.3. Intervention process

Detailed intervention packages outline the steps to be taken while implementing this trial was developed. Training manuals and guiding tools for health care providers and school administrators were also developed. Initiation orientation for schools and health administrators and detailed training for HCP, data collectors, and supervisors was conducted. The intervention was launched after the pretest in both study arms. The intervention was launched on November 2020 and ended on May 2021 after a 6-month period (Table 1).

**Table 1:** Descriptions of school-linked life skills intervention program for adolescents among trial schools in Gorodola district Guji zone, Ethiopia (November 2020 to May 2021).

**Intervention**	**Contents**	**# of Session**	**Goals**	**Training methods**
Life skills for self-understanding and self-management	Self-awareness	6	Understand about oneself	Group learning, scenario, control over, and focused dialogue
	Copy with stress	6	Learn and control the stress	Lecture, role-play scenarios, discussion, and group learning
	Copy with emotion	6	Learn and control powerful feelings	Watch the videos, read, and dialogue scenarios' discussion during group learning
Life skills for knowing and living with others	Effective communication	6	Properly express oneself	Lecture, role play, discussion, group learning, and worksheets
	Interpersonal relationship	6	Learn and relate in positive ways with people	Lecture, role play scenarios, discussion, and group learning
	Empathy	6	Learn and exercise to understand the thoughts and feelings of others	Watch the videos in classes, discussions, and group learning
Life skills for dealing with issues and problems	Critical thinking	6	Lean and think analytically	Lecture, role-play scenarios, discussion, and group learning
	Creative thinking	6	Learn and produce something novel	Lectures, role-play scenarios, interactive discussions, and written worksheets
	Decision making	6	Learn and conclude	Group learning and role-play scenarios assist how to solve
	Problem-solving	6	Learn and practice problem-solving techniques	
Age-relevant SRH education	Nature of adolescents	4	Understand changes and nature of adolescent growth	Group learning, lecture, pictures, and focused dialogue
	RHR and wellbeing	4	Aware SRH and wellbeing	Lecture, role-play scenarios, and group discussion
	Healthy sexual behavior and relations	6	Awareness of healthy sexual behaviors and relationship	Watch videos, read and dialogue scenarios and worksheet
	Adolescents SRH Problems	4	Learn common causes of adolescents SRH problems	Group learning, lecture, group discussion, show posters

#### 2.4.4. Tools and techniques

**Figure 3:**
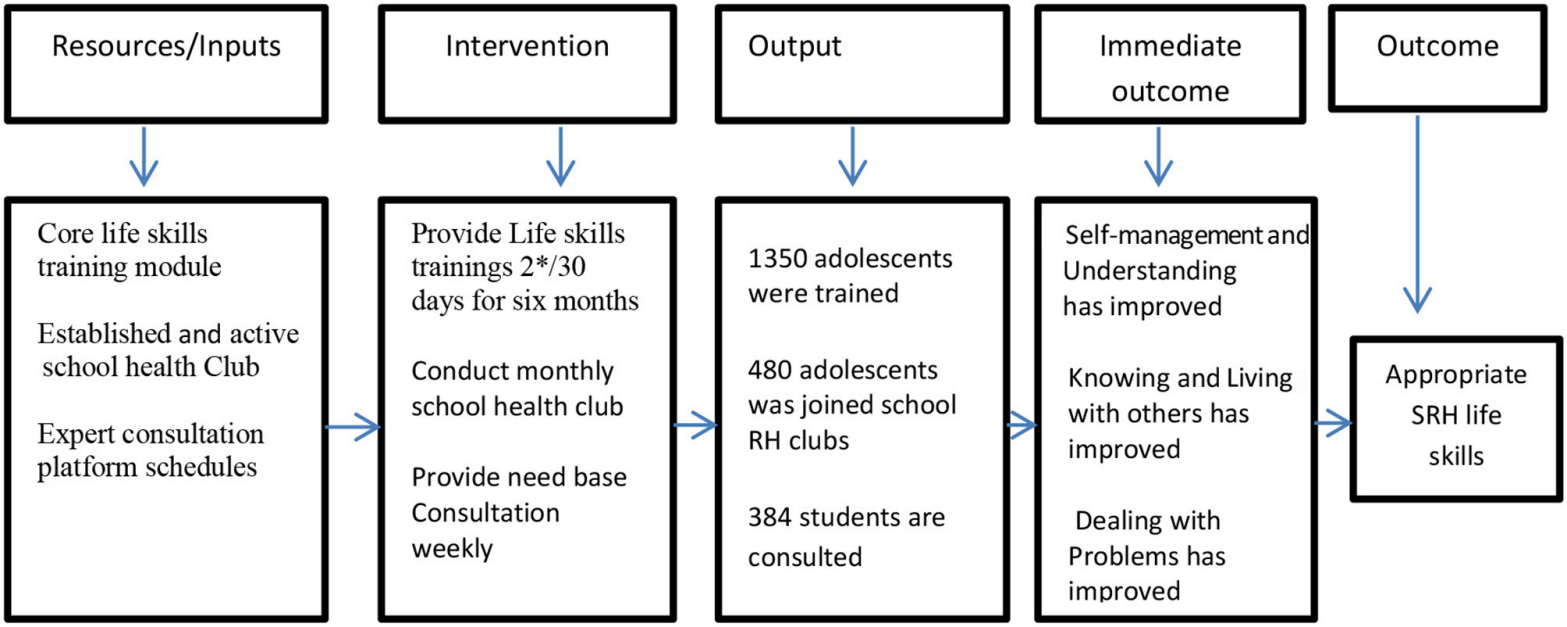
Adolescents' life skills intervention logic model framework adopted from Smith et al. (27). High school adolescents of Guji Zone, South Ethiopia, February 2022.

A pretested structured questionnaire was used to collect the data. The questionnaire was developed after reviewing relevant literature (23, 24). The English version questionnaire was translated into Oromiffa (the local language of the study area). The Oromiffa version was printed and shared with data collectors. The baseline and end-line studies used similar questionnaires to collect data and conducted face-to-face interviews. This study employed a logic model as a guiding framework (25). The assumption was to present intended intervention among the study group to produce an outcome of interest through interconnected relationships between the resources, activities outputs, and intended outcomes (26, 27) (Figure 3).

#### 2.4.5. Variables and measurement

This trial aimed to determine the effect of school-linked adolescents' friendly life skills intervention on improving SRH life skills. In the end-line survey, SRH life skills were compared between the intervention and control clusters. Moreover, factors that enhance life skills development were investigated. It is worth noting that SRH is of great importance. SRH life skills were identified as the outcome variable. High school sessions were taken as a clustering variable, and then, the independent variables were categorized as personal-level, behavioral-level, and environmental-level variables. Hence, four groups of life skill development with supposed cognitive mediators were used: information, self-awareness, critical thinking and self-esteem, and empathy. We analyzed and measured the cognitive variables influencing behavior changes in adolescents' life skills development. We used 27 items to measure appropriate SRH life skills in this study: agree/one had appropriate information, self-awareness, critical thinking and self-esteem, and empathy, and disagree/zero if not. The levels of SRH life skills were categorized using mean scores; thus, a life skills score above the mean is considered the appropriate SRH life skills (28).

#### 2.4.6. Data management and analysis

Following the field data collection, the data were entered into Epidata software version 3.1 and were analyzed using SPSS version 23.0. Data were checked for any missed values and outliers. Proportions, the mean, and standard deviations were employed to depict descriptive data. Item elimination was based on factor loading criteria. Similarly, the status of the SRH life skills between the intervention and control arms was compared using the chi-squared association test. Factors enhancing SRH life skills were identified using a bivariate analysis to test the relationship between each independent and outcome variables. This study employed a generalized linear model (GLM) (29). The presence of cluster-level variability influencing SRH life skills was tested by the intercept-only model and intraclass correlation coefficient (ICC) (30, 31). Moreover, the disparity among the clusters was measured using the median odds ratio (MOR). The ICC was performed to calculate the proportion of total variance in the outcome attributed to the area level, whereas MOR was used to calculate unexplained cluster heterogeneity (32, 33).

The model fitness for the multi-level model was tested using the log-likelihood ratio (LR). Four models were created during the analysis. The first model was used to determine the effect of cluster variation on SRH life skills. The second model was used to determine individual-level variables, the third model was used for behavioral-level variables, and the fourth model was used for environmental-level variables. We performed a bivariate analysis first, and only the variables with a *p* < 0.25 were included in the second, third, and fourth models. However, only variables with a *p* < 0.05 were included in the final model. The statistical significance was defined with a *p* < 0.05. The strength of association and significance level were identified with AOR and 95% CI, respectively (34, 35).

The mean difference in adolescents' life skills between the trial and control arms was compared using paired sample independent *t*-tests (35, 36). Factor analysis and reliability tests were used to check the internal consistency of data and model fitness (37, 38).

## 3. Results

### 3.1. Sociodemographic characteristics

*Baseline:* A total of 773 (98%) adolescents, aged 14–19 years, from four high schools and 30 clusters in the pastoral districts of Gorodola and Wadara were recruited. Approximately 384 (49.7%) were from intervention clusters, and 389 (50.3%) were from control clusters. The mean age in the experimental arms was 17.34 (±SD = 1.37) compared with 17.26 (±SD = 1.3) in the control group. The mean learning level of participants in the trial arm was 10.2 (±SD ±1.269) compared with 10.68 (SD = 0.75) in the control arms (Table 2).

**Table 2:** Sociodemographic characteristics of study participants disaggregated by baseline and end line, intervention and control clusters, East Guji zone, south Ethiopia, November 2020 to May 2021 (*n* = 1,532).

**Variables**	**Category**	**Baseline (*N* = 788, *n* = 773)**				**End line (*N* = 788, *n =* 759)**			
		**Intervention** ***n** =* **384**		**Control** ***n** =* **389**		**Intervention** ***n** =* **375**		**Control** ***n** =* **384**	
		**Freq**.	**%**	**Freq**.	**%**	**Freq**.	**%**	**Freq**.	**%**
Residence	Urban	145	38%	195	50%	162	43%	202	53%
	Rural	239	62%	194	50%	213	57%	182	47%
Age	Middle-aged adolescents	119	31%	109	28%	129	34%	116	30%
	Late-aged adolescents	265	69%	280	72%	246	67%	268	70%
Sex	Male	256	67%	203	52%	231	62%	201	52%
	Female	128	33%	186	48%	144	38%	183	48%
Marital status	Single	347	90%	360	93%	345	92%	357	93%
	Married	34	9%	25	6%	30	8%	16	4%
Education	Grades 9 and 10	196	51%	190	49%	204	54%	228	59%
	Grades 11 and 12	188	49%	199	51%	171	46%	156	41%
Religions	Protestant	227	59%	138	36%	246	66%	176	46%
	Muslim	94	25%	170	44%	90	24%	162	42%
	Orthodox	18	5%	57	15%	10	3%	32	8%
	Others	45	12%	24	6%	29	8%	14	4%

*End line:* Data were collected from 759 (93.3%) adolescents who participated in 30 school sessions within similar pastoral districts. Among them, 375 (47.58%) were from intervention clusters and 384 (48.73%) from control clusters. The mean age was 17.26 (±SD = 1.511) in the experimental group compared with 17.28 (±SD = 1.398) in the control group. The mean learning level of the trial arm was 9.93 (±SD ± 0.997) compared with 10.08 (SD ± 0.933) in the control arm (Table 2).

### 3.2. Comparison of two “populations” mean

Paired-sample independent *t*-test analysis results showed that the mean life skills among adolescents, recruited as the test group (M = 0.7049, SD = 0.16385, *N* = 375), were significantly different from the control arm (M = 0.5755, SD = 0.15775, *N* = 384), t (11.321), *P* < 0.001. Therefore, the null hypothesis was rejected at 95% CI on the difference between the two population means using an independent *t*-test distribution with 374° of freedom at 95% CIs (0.10692, 0.15187).

### 3.3. Status of adolescents' SRH life skills

*Baseline:* The baseline survey findings showed that the proportion of adolescents with SRH life skills was 150 (39.1%) among the intervention and 220 (56.6%) among the control clusters. The mean scores of SRH matter life skills were 0.5198 among the experimental arms and 0.6031 among control clusters at a *p* < 0.001; 95% CI= (−0.1 to −0.05855), with a negative mean difference of −0.08324) (Table 3).

**Table 3:** Mean Scores, proportions of SRH life skills, and comparison of life skills mean scores among high schools adolescent, Guji zone, Ethiopia, February 2022.

**Variables**	**Baseline**				**End line**			
	**Intervention clusters (*****n** =* **384)**	**Control clusters (*****n** =* **389)**	**P (95% CI)**	**Mean Diff**.	**Intervention clusters (*****n** =* **375)**	**Control clusters (*****n** =* **384)**	**P (95% CI)**	**Mean Diff**.
Trial and control Mean scores	0.5198	0.6031	*P <* 0.001; 95% CI = (−0.1 −0.059)	−0.083	0.7049	0.6225	*P <* 0.001; 95% CI=(0.06–0.1)	0.0824
Trial clusters' pre-post mean scores	0.5198		*P <* 0.001, 95% CI (−0.1 to −0.057)		0.7049		*P <* 0.001 (0.157 to 0.21)	0.18179
Control clusters pre-post mean score		0.6031	*P <* 0.001, 95% CI (−0.1 to −0.057)			0.6225	*P <* 0.1 (−0.00354, 0.04)	0.01854
The proportion of SRH to life skills.	150 (39.1%)	220 (57%)		−18%	252 (67%)	234 (61%)		6.3%

*End line:* The end-line findings showed that the proportion of adolescents with improved SRH life skills was 252 (67.2%) among the intervention cluster and 234 (60.9%) among the control cluster. However, after the interventions, the mean life skills scores showed that the test group had more progressive scores than the control arm with a statistically significant difference of 70.49% and 62.25% among the control clusters at *P* < 0.001; 95% CI=0.06 to 0.1, with a positive mean difference of 0.0824) (Table 3).

### 3.4. Effect of the intervention

**Figure 4:**
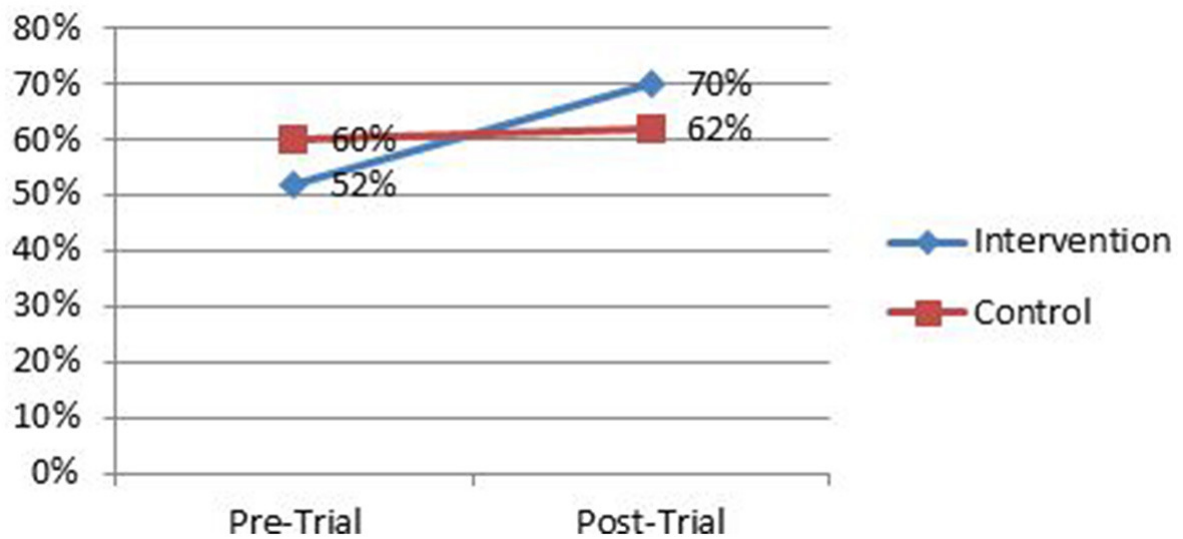
Comparison of mean life skills disaggregated by baseline and end line and intervention and control arms among high school adolescent students, East Guji zone, Ethiopia, November 2020 to May 2021.

Effectiveness of a school-linked life skills intervention program (LSIP) is improved SRH life skills. The paired independent t-test analysis revealed a statistically significant mean difference in life skills development between the intervention and control arms [8.2%, 95% CIs (0.15657 to 0.2) *P* < 0.001] over a period of time. Compared to the control clusters group, the proportion of adolescents with appropriate SRH life skills was significantly higher in the intervention clusters 375(70.5%) vs. 384(62%) *P* < 0.001 (Table 3, Figure 4).

#### 3.4.1. Factors contributing to life skills development

The results from the Generalized Linear Model (GLM) analysis revealed that, among the individual-level variables, training on the life skills development manual was significant (model 2). Among the behavioral-level variables, the confidence to make safe and informed decisions and the necessary life skills to deal with SRH issues were significant (model 3). Regarding environmental-level variables, the district and residence place receiving school-linked life skills intervention was significant (model 4). However, only the variables at the individual level and the behavioral level became significant in the final model.

Adolescents who were trained on the SRH life skills showed a significant improvement in mean proper life skills score compared to the control arm at unstandardized coefficient (β = 1.915, 95% CI: 1.411–2.418). Adolescents in the intervention clusters (β = 1.999, 95% CI: 1.562–2.436) were predicted to be more confident to make safe and informed decisions than those in the control clusters. We found that adolescents in the intervention clusters were more likely to possess the necessary life skills to deal with SRH issues (β = 1.66, 95% CI: 1.233–2.087) than those in the control cluster. This study showed that adolescents in the intervention clusters (β = 1.563, 95% CI: 0.994–2.132) were more likely to understand others' feelings than those in the control clusters. Adolescents who identified their vision, abilities, and short- and long-time goals (β = 2.069, 95% CI: 1.562–2.576) were more predictable to have appropriate SRH life skills than others (Table 4). The Pearson chi-squared analysis revealed significant association between outcome variables and school-linked SRH life skills intervention (84.388, *P* < 0.001). Similarly, the intercept of generalized linear model analysis revealed that 94.5% (ICC: 94.5%: *P* < 0.001) of the variation was attributable to the cluster level.

**Table 4:** Sexual and reproductive health life skills progress-related factors, Guji, zone, South Ethiopia, February 2022 (*n* = 759).

**Variables**	**Control**		**Intervention**		**X^2^ test**	**Coefficient of beta (β)**	***P*-value**
	**Frequency**	**%**	**Frequency**	**%**			
**Ever trained in life skills?**							
Yes	64	16.70%	185	49.30%	55.6 1.915	1.915	0.001
No	320	83.20%	190	50.70%			
**Confident enough to make safe and informed decision**							
Yes	198	51.60%	245	65.30%	80.518	1.999	0.001
No	186	48.40%	130	34.70%			
**Coping with stress and emotion are core life skills that help for self-understanding**							
Yes	173	45.10%	248	66.10%	23.828	1.046	0.001
No	211	54.90%	127	33.90%			
**Identified vision, abilities, and short- and long-time goals**							
Yes	246	64.10%	292	77.90%	64.059	2.069	0.001
No	138	35.90%	83	22.10%			
**Understand others' feelings without sharing their interest**							
Agree	99	25.80%	321	85.60%	28.953	1.563	0.001
Disagree	285	74.20%	54	14.40%			
**Necessary life skills to deal with SRH issues**							
Yes	169	44%	255	68%	58.123	1.66	0.001
No	215	56%	120	32%			

## 4. Discussion

The findings indicated that the intervention program significantly improved adolescents' SRH life skills in the study areas and that life skills intervention packages were an interactive educational model to shape attitudes and develop SRH life skills. In this present study, both individual-level factors and behavioral-level factors predicted significant improvements in life skills development than environmental-level factors. This implied that, once adolescents had been trained in school-linked life skills intervention, their SRH life skills had been greatly influenced by factors that operate at the individual and behavioral level. The results of this study showed that school-linked life skill intervention improved adolescents' life skills with increased self-awareness, safe and informed decision-making, identifying their vision, abilities, and goals, understanding others' feelings, and possessing skills to deal with SRH issues through interactive learning and communicative skills. These results are in line with school-based study programs in Thailand (24), India (6), and the Netherlands (25). In Ethiopia, these findings are also similar to Baird et al. (14) which showed significant progress in the knowledge of menstrual hygiene skills and reproductive health issues (16). Moreover, in India, the outcome also aligns with the findings of Sukumar et al. (39), which showed significantly improved life skill scores (39).

The statement, “Our research showed that individual-level and behavioral-level factors had predicted more improvement in life skills development than environmental-level factors,” suggests that the government could focus more on individual- and behavioral-level factors. Moreover, the finding demonstrated that life skills in the trial and control arms had a negative mean difference. However, after the intervention, the mean differences in life skills between the intervention and control arms significantly improved. The mean difference showed that the intervention program had significantly changed the intervention arm.

Moreover, the mean difference within the trial arms was higher in pre-trial outcomes with significant positive changes. The Pearson chi-squared analysis revealed that significant association between the outcome variable and school-linked SRH life skills intervention. Compared to the control arm, adolescents in the intervention clusters developed proper life skills. This result implied that school-linked SRH life skills intervention was significantly correlated with appropriate SRH life skills development. This suggestion was communicated with proposals and school-linked life skills intervention trial studies (8, 12, 14, 15, 39–41). This demonstrated that adolescents exposed to the life skill training had appropriate SRH life skills.

In the end study, the proportion of adolescents with life skills increased from 370 (47.9%) to 382 (50.3%). The change in proportion was 2.4% and may look small. However, the arithmetic means at baseline of 0.56 increased to 0.64 at the end-line. This finding aligns with the results of Magalhaes et al. (9) from Portuguese, where 50.7% of adolescents exposed to a school-linked life skills intervention program demonstrated improved social skills. Similarly, in Spain, Gasol et al. (10) reported a rate of 48%. However, our results indicated lower scores compared to the study conducted by Pradeep et al. (1) in India, where 91.6% of participants showed improved life skills awareness. However, the findings exceed those of Malango and Hegena (11) in Ethiopia, where the rate was 25.7%, and Tesema et al. (12), also in Ethiopia, which reported a rate of 24.5%. According to the findings, life skills are projected to increase by 1.915 for each additional training session provided on each day during the school-linked life skills intervention program. These findings, consistent with studies conducted in Thailand, India, and Ethiopia (14, 15, 42), implies that experiential learning programs tend to be more effective than non-trial learning methods.

## 5. Conclusion and recommendation

In conclusion, the school-linked life skills intervention program has significantly increased appropriate SRH life skills among high school adolescents in the East Guji zone, Ethiopia. Based on the results, we reject the null hypothesis due to the significant difference between the means of the two populations, as determined by an independent *t*-test with 374 degrees of freedom at a 95% confidence interval (0.106, 0.152). Providing a school-linked life skills intervention program and instilling confidence to make safe and informed decisions were the most significant predictors for developing appropriate SRH life skills.

The findings suggest that the MoH in Ethiopia should strengthen school-linked life skills intervention programs within adolescent-friendly health settings, with a particular emphasis on fostering self-awareness to improve life skills among school adolescents. Therefore, health sector authorities, health facilities, school administrators, and relevant stakeholders should consider adopting a school-linked life skills intervention approach.

### 5.1. Strength and limitations

We employed a double-blind approach in this study. Consequently, both adolescents and data collectors were unaware of the intervention, and they were assigned to different study arms. Furthermore, the study was conducted in collaboration with public schools linked to nearby health facilities that provided the interventions. The data collectors were also kept unaware of whether they were in the intervention or control arms. The study compared baseline and end-line outcomes of interest between the intervention and control arms. Moreover, high schools/clusters were identified and recruited before randomization.

Furthermore, this study was exclusively conducted in public schools; hence, the findings cannot be generalized to private school adolescents. Additionally, the trial period was relatively small, spanning from November 2020 to May 2022. Therefore, the impact might not be evaluated. Henceforth, conducting a long-term experimental study, supported by qualitative research, is crucial for further investigations.

## Data availability statement

The original contributions presented in the study are included in the article/supplementary material, further inquiries can be directed to the corresponding author.

## Ethics statement

The studies involving human participants were reviewed and approved by the Jimma University (Ref. no. IHRPCA/721/202, date 17/08/2020) and the Bureau of Regional Health, Oromia (Lakk/Ref. no. BEFO/MBTF/2081, date 27/01/2013, ETC. or 07/10/2020). Earlier in the in-depth interview and focus group discussion (FGD), all study participants gave verbal informed consent. The qualitative data collection was completed in November 2020. Written informed consent to participate in this study was provided by the participants' legal guardian/next of kin.

## Author contributions

GG is the principal investigator, conceptualizing the research idea, drafting the proposal, collecting data, analyzing it, writing up the whole manuscript, and communicated with co-authors and organized their works in the manuscript. MA drafted the proposal and wrote the methodology part. SG consulted and participated in the data analysis process and arranged the order of the manuscript. Furthermore, NA consulted in the technical language edition and wrote the results part. All authors significantly contributed from protocol development to the manuscript writing process.
